# Mechanical properties, *in vitro* corrosion and biocompatibility of newly developed biodegradable Mg-Zr-Sr-Ho alloys for biomedical applications

**DOI:** 10.1038/srep31990

**Published:** 2016-08-24

**Authors:** Yunfei Ding, Jixing Lin, Cuie Wen, Dongmei Zhang, Yuncang Li

**Affiliations:** 1 School of Engineering, RMIT University, Melbourne, Victoria 3001, Australia; 2 Department of Materials Science and Engineering, Jilin University, Changchun, Jilin 130025, China; 3 Institute for Frontier Materials, Deakin University, Geelong, Victoria 3217, Australia

## Abstract

Our previous studies have demonstrated that Mg-Zr-Sr alloys can be anticipated as excellent biodegradable implant materials for load-bearing applications. In general, rare earth elements (REEs) are widely used in magnesium (Mg) alloys with the aim of enhancing the mechanical properties of Mg-based alloys. In this study, the REE holmium (Ho) was added to an Mg-1Zr-2Sr alloy at different concentrations of Mg1Zr2SrxHo alloys (x = 0, 1, 3, 5 wt. %) and the microstructure, mechanical properties, degradation behaviour and biocompatibility of the alloys were systematically investigated. The results indicate that the addition of Ho to Mg1Zr2Sr led to the formation of the intermetallic phases MgHo_3_, Mg_2_Ho and Mg_17_Sr_2_ which resulted in enhanced mechanical strength and decreased degradation rates of the Mg-Zr-Sr-Ho alloys. Furthermore, Ho addition (≤5 wt. %) to Mg-Zr-Sr alloys led to enhancement of cell adhesion and proliferation of osteoblast cells on the Mg-Zr-Sr-Ho alloys. The *in vitro* biodegradation and the biocompatibility of the Mg-Zr-Sr-Ho alloys were both influenced by the Ho concentration in the Mg alloys; Mg1Zr2Sr3Ho exhibited lower degradation rates than Mg1Zr2Sr and displayed the best biocompatibility compared with the other alloys.

Biodegradable implants used in the human body are essential to provide adequate mechanical integrity and a suitable corrosion rate before sufficient tissue healing^1^. Biomaterials should also have acceptable biocompatibility and minimal deleterious effects on organisms^2^. Importantly, the components of biomaterials should be not only biocompatible, but also promote the growth and healing of tissue^3^. Among the metallic biomaterials for hard tissue engineering, magnesium (Mg) alloys are receiving increasing attention as promising biodegradable materials for orthopaedic applications because of their remarkable advantages^4^. Although significant progress has been achieved in the development of biodegradable Mg alloys, some issues, such as rapid corrosion with generated hydrogen gas, weakened mechanical integrity over time and potential toxicity of their components, still restrict their application. Hence, it is crucial to develop Mg alloys with bio-friendly alloying elements and enhanced biocorrosion resistance^5^,^6^.

Recent studies have found that rare earth elements (REEs) exhibit many desirable advantages in Mg alloys, such as improved corrosion resistance and enhanced mechanical properties^7^,^8^,^9^,^10^,^11^,^12^. REE-containing Mg alloys are the most successful biodegradable alloys for biomedical applications; e.g. WE43 has been successfully used in clinical application^13^. Among REEs, Ho (>0.5 wt. % hereafter) has been used to enhance the tensile properties of Mg-Zn-Al due to the reduction of the petal-like phase^14^ and Ho also displays potential effects on grain refinement, resulting in significant improvement in strength and plasticity^15^. However, in view of the performance of Ho in relation to biocompatibility, a limited number of studies made comparisons under identical experimental conditions^16^,^17^. Although Mg-REE-based alloys for cardiovascular applications have been used in clinical trials, for the bulk of Mg-REE alloys in orthopaedic applications, concerns about the biosafety and usage of REEs have been be raised^18^, as there is no consensus on their safe dosage^4^. Therefore, it is essential to understand the roles of REEs in the microstructure, mechanical properties, corrosion and biocompatibility of Mg alloys.

A recent breakthrough in the development of Mg alloys for orthopaedic applications was achieved by a group led by Li^19^, leading to a series of Mg-Zr-Sr alloys with enhanced corrosion resistance compared to cast pure Mg and excellent biocompatibility in the form of support of cell adhesion and spreading. In detail, the addition of zirconium (Zr) to Mg alloys can significantly refine the grain size, which benefits the mechanical properties and corrosion resistance^20^,^21^. The alloying element strontium (Sr) significantly enhances the replication of preosteoblastic cells, and even stimulates bone formation^22^,^23^,^24^. Considering the benefits of Zr, Sr and Ho in enhancing biocompatibility, corrosion and mechanical properties, a new series of Mg1Zr2SrxHo alloys (x = 1, 3, 5%) have been developed in this study to satisfy the urgent and exclusive requirements for hard tissue engineering.

## Results

**Figure 1:**
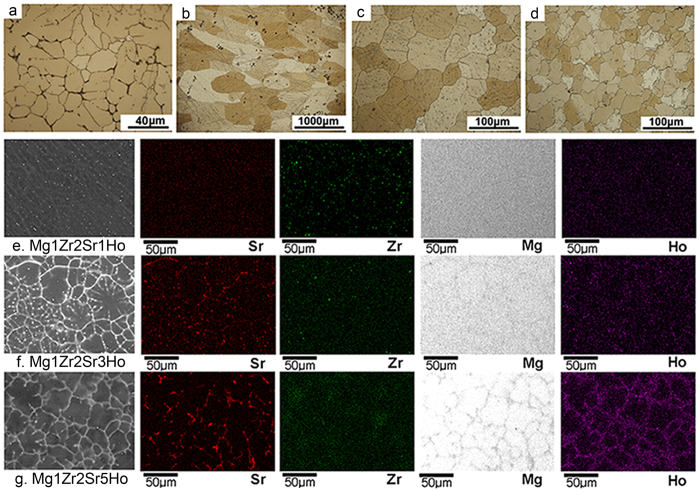
Microstructures of Mg-Zr-Sr-Ho alloys: (**a**) Mg1Zr2Sr, (**b**) Mg1Zr2Sr1Ho, (**c**) Mg1Zr2Sr3Ho, (**d**) Mg1Zr2Sr5Ho; and EDX mapping of the alloys: (**e**) Mg1Zr2Sr1Ho, (**f**) Mg1Zr2Sr3Ho, (**g**) Mg1Zr2Sr5Ho.

The microstructures of the Mg-Zr-Sr-Ho alloys are shown in Fig. 1a–d. The grain size of Mg1Zr2Sr1Ho (200~1000 μm) is relatively larger than that of Mg1Zr2Sr (20~50 μm) and the other Mg-Zr-Sr-Ho alloys (20~80 μm). However, with the increasing addition of Ho (from 1% to 5%), the grain size dramatically decreased in Mg1Zr2Sr3Ho and Mg1Zr2Sr5Ho, compared to Mg1Zr2Sr1Ho. Also, some tiny black particles can be seen in Mg1Zr2Sr and Mg1Zr2Sr1Ho, while Mg1Zr2Sr3Ho and Mg1Zr2Sr5Ho showed homogeneous structures without the appearance of black particles. The XRD patterns of the Mg-Zr-Sr-Ho alloys are shown in Fig. S1, displaying the characteristic crystalline structure of Mg-Zr-Sr-Ho alloys, including Mg, Zr, Mg_17_Sr_2_ and Mg-Ho phases. It is evident that the intensity of the peaks for the Mg_17_Sr_2_ phase slightly decreased with increasing Ho addition in the Mg1Zr2SrxHo alloys. Two different Ho-containing phases, that is, MgHo_3_ (JCPDS No. 03-065-7200) and Mg_2_Ho (JCPDS No. 04-002-0737), were detected in Mg1Zr2Sr3Ho and Mg1Zr2Sr5Ho, suggesting that the Ho addition to Mg1Zr2Sr led to the formation of the intermetallic Mg-Ho phases. In addition, the MgHo_3_ and Mg_2_Ho phases exhibited different intensities in the Mg-Zr-Sr-Ho alloys. The volume fraction of Mg_2_Ho in Mg1Zr2Sr3Ho was much higher than in Mg1Zr2Sr1Ho and Mg1Zr2Sr5Ho (Fig. 1 and Fig. S1); whereas the intensity of MgHo_3_ increased dramatically with the increase in Ho addition to Mg1Zr2Sr5Ho, indicating a higher quantity of MgHo_3_ than in Mg1Zr2Sr3Ho and Mg1Zr2Sr1Ho. Energy-dispersive X-ray spectroscopy (EDX) mapping of the elemental distribution over the Mg-Zr-Sr-Ho alloys (Fig. 1e,f) reveals that the intermetallic phases of Mg_17_Sr_2_, MgHo_3_ and Mg_2_Ho were mainly distributed along grain boundaries, and the signals for Sr were surpassed by those of the Mg-Ho phases, in agreement with the XRD analysis and confirming that the Ho additions to the Mg-Zr-Sr-Ho alloys influenced the microstructures of the alloys.

**Figure 2:**
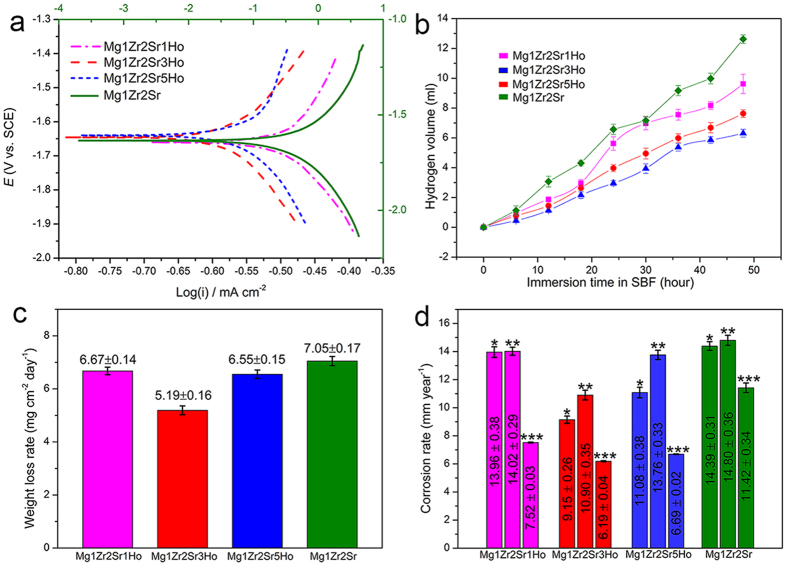
Corrosion behaviour of Mg-Zr-Sr-Ho alloys: (**a**) polarisation curves; (**b**) hydrogen evolution; (**c**) weight loss rates; (**d**) calculated corrosion rates (*calculated based on hydrogen evolution, **calculated based on weight loss, ***calculated based on polarisation tests).

Figure 2a shows the Tafel curves of the Mg-Zr-Sr-Ho alloys after immersion in simulated body fluid (SBF) for 2 h at 37 ± 0.5 °C. As can be seen, Mg1Zr2Sr3Ho presented the lowest current density compared with Mg1Zr2Sr and the other Mg-Zr-Sr-Ho alloys. In contrast, Mg1Zr2Sr1Ho exhibited the highest current density among the Mg-Zr-Sr-Ho alloys, indicating the lowest corrosion resistance in SBF. Compared to Mg1Zr2Sr1Ho and Mg1Zr2Sr3Ho, Mg1Zr2Sr5Ho displayed a higher potential, indicating that MgHo_3_ and Mg_2_Ho phases (XRD, Fig. S1) have a different potential compared with the Mg matrix, and the increased volume fraction of the intermetallic phases created a higher open-circuit potential for the Mg-Zr-Sr-Ho alloys.

Analogous to the polarisation test, which represents corrosion behaviour at a select point of time, hydrogen evolution is a reliable method to assess the average corrosion rates of Mg alloys^25^. The rate of generated hydrogen gas against immersion time in SBF is shown in Fig. 2b. After washing using a 200 g L^−1^ chromic acid solution, the weight loss rate was measured and its results are listed in Fig. 2c. The fitting results of polarisation tests, and the corrosion rates based on polarization test, hydrogen evolution and weight loss are summarised in Table 1 and Fig. 2d, respectively. As can be seen, although the values of the corrosion rate based on these various assessment methods are different, the results reveal that Mg alloys with different Ho additions suffered different attacks from SBF in the increasing order of Mg1Zr2Sr3Ho < Mg1Zr2Sr5Ho < Mg1Zr2Sr1Ho < Mg1Zr2Sr, which suggests that the addition of Ho to Mg1Zr2Sr is beneficial to corrosion resistance and that Mg1Zr2Sr3Ho is relatively stable compared to the other Mg-Zr-Sr-Ho alloys.

The dissolution of Mg in a physiological environment generally involves an electrochemical reaction with water and produces magnesium hydroxide (Mg(OH)_2_) and hydrogen gas (H_2_)^5^,^26^: Mg + 2H_2_O → Mg(OH)_2_ + H_2_. The generated Mg(OH)_2_ at the initial stage provides temporary protection for the Mg alloys, retarding further corrosion^26^. Thus, it is essential to identify the corrosion products on the surfaces of the Mg alloys. It can be seen that the corrosion products on the Mg-Zr-Sr-Ho alloys mainly contained MgO and Mg(OH)_2_ (Fig. S2). Specifically, the intensities of the MgO and Mg(OH)_2_ peaks in Mg1Zr2Sr3Ho are higher than those of the other alloys, indicating better protection provided by the corrosion products (Fig. S2b).

**Figure 3:**
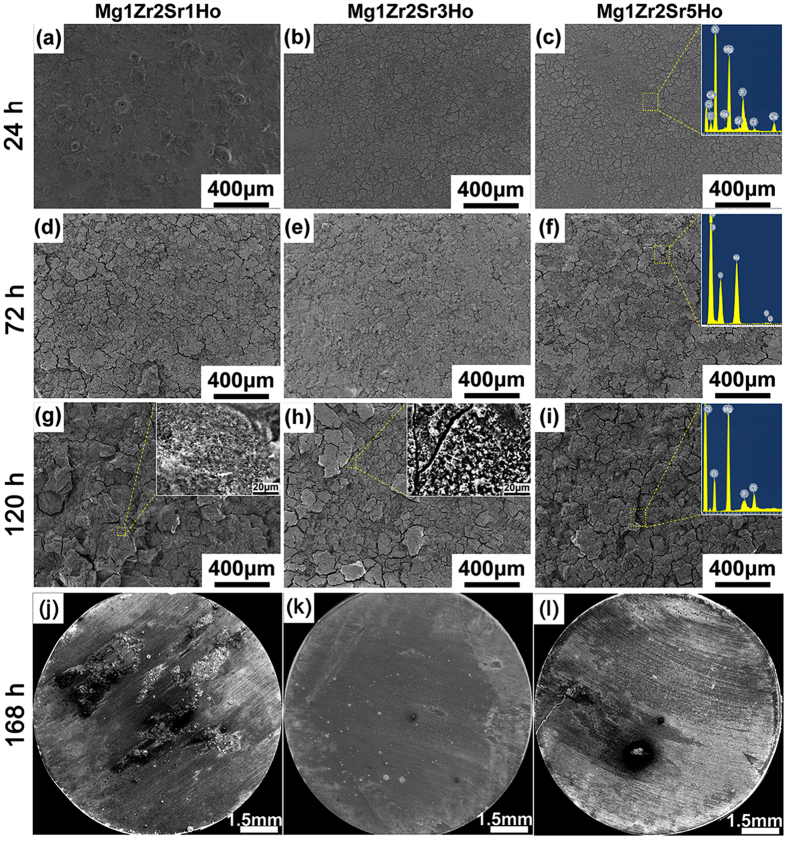
Corrosion morphologies of Mg-Zr-Sr-Ho alloys after immersion in SBF for: (**a**–**c**) 24 h, (**d**–**f**) 72 h, (**g**–**i**) 120 h; and (**j**–**l**) overall corrosion morphologies of Mg alloys after 168 h immersion in SBF.

Figure 3 shows the corrosion morphologies of the Mg-Zr-Sr-Ho alloys after immersion in SBF for various time periods (24 h, 72 h, and 120 h). It can be seen that the surface of each alloy was covered with a layer of corrosion product after immersion in SBF for 24 h (Fig. 3a–c). The corrosion products on Mg1Zr2Sr3Ho and Mg1Zr2Sr5Ho exhibited a similar corrosive pattern and a loose structure, indicating a lower corrosion rate than that of Mg1Zr2Sr1Ho. EDX of the selected region of Mg1Zr2Sr5Ho (Fig. 3c) revealed that the corrosion products were mainly composed of magnesium, oxygen, chlorine and calcium. However, chlorine- and calcium-containing phases were not identified in XRD patterns (Fig. S2), which might be explained by the low quantity on the surfaces.

The corrosion morphologies of the Mg-Zr-Sr-Ho alloys after immersion in SBF for 72 h are shown in Fig. 3d–f. Compared to Mg1Zr2Sr1Ho and Mg1Zr2Sr5Ho, Mg1Zr2Sr3Ho still maintained its surface integrity and a relatively dense structure (Fig. 3e). EDX investigation of selected region of Mg1Zr2Sr5Ho showed that the corrosion products consisted of chlorine, oxygen and magnesium (Fig. 3f). It is evident that the chlorine from SBF deteriorated the corrosion products (MgO and Mg(OH)_2_)^27^, and thus the corrosion rate of Mg1Zr2Sr5Ho subsequently increased due to the absence of temporary protection by the corrosion products.

After 120 h of immersion in SBF, the Mg-Zr-Sr-Ho alloys were further deteriorated. At highly magnified SEM image of Mg1Zr2Sr3Ho demonstrates that the corrosion products broke down into a network structure (Fig. 3h). In contrast, a rough surface can be observed on Mg1Zr2Sr1Ho, indicating large quantities of the Mg matrix dissolved into SBF (Fig. 3g). For the Mg1Zr2Sr5Ho alloy the surface was further deteriorated, as indicated by the increased signal of Cl^−^ (Fig. 3i).

The overall surfaces of the Mg-Zr-Sr-Ho alloys after immersion in SBF for 168 h exhibited differently corroded morphologies, indicating different corrosion behaviours of the alloys (Fig. 3j–l). The surface of Mg1Zr2Sr3Ho still retained its overall integrity and relatively uniform corrosion, demonstrating the highest corrosion resistance in SBF (Fig. 3k), while grooves were formed on the surface of Mg1Zr2Sr1Ho by severe attacks from SBF ions, and the regions undergoing severe attacks were much wider and deeper than in other Mg-Zr-Sr-Ho alloys (Fig. 3j). As for Mg1Zr2Sr5Ho, the majority of the surface was covered with white corrosion products; pits are also visible in some regions and a long corroded crack extends to the border (Fig. 3l).

**Figure 4:**
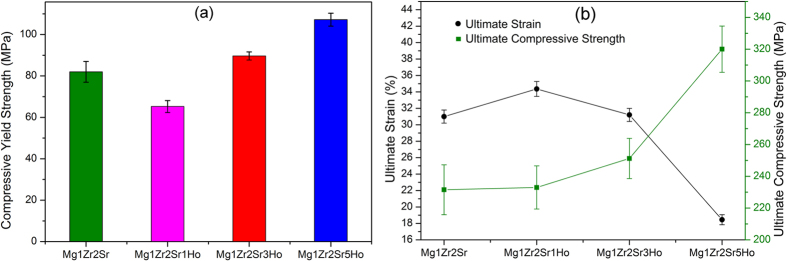
Mechanical prosperities of Mg-Zr-Sr-Ho alloys: (**a**) compressive yield strength, (**b**) ultimate strain and ultimate compressive strength.

Compressive testing has been widely used to evaluate the mechanical properties of Mg alloys, as it possesses the advantage of simple operation by loading circular cylinder samples between rigid platens which effectively constrain the movement of the samples at the loaded interfaces^28^,^29^. Figure 4 shows the yield strength, the ultimate compression strength and the strain of the Mg-Zr-Sr-Ho alloys. It can be seen that the yield strength of Mg1Zr2Sr1Ho was lower than that of Mg1Zr2Sr (Fig. 4a), however, it increased with the increase in the addition of Ho. Similarly, Mg1Zr2Sr3Ho and Mg1Zr2Sr5Ho exhibited higher compressive yield strengths than those of Mg1Zr2Sr and Mg1Zr2Sr1Ho (Fig. 4b). The ultimate compressive strength of the Mg-Zr-Sr-Ho alloys increased with the increase in Ho addition, suggesting that the addition of Ho enhances compressive strength (Fig. 4b). It is worth noting that Mg1Zr2Sr1Ho showed the highest ultimate strain (~34%) and the ultimate strain decreased with the increase in the addition of Ho. It can be concluded that the concentration of Ho in Mg-Zr-Sr-Ho alloys has a significant effect on their mechanical properties.

**Figure 5:**
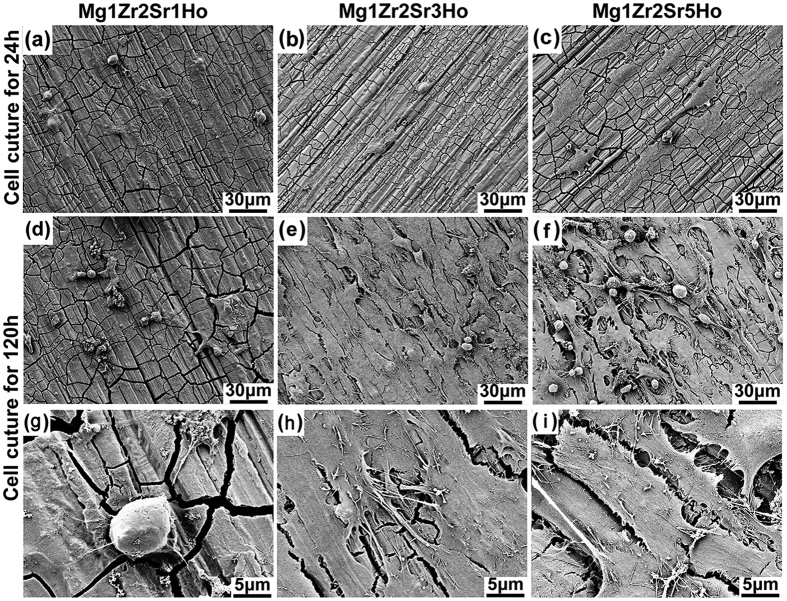
Cell adhesion and cell numbers on the Mg-Zr-Sr-Ho alloys after cell culture for various periods: (**a**–**c**) 24 h, (**d**–**f**) 120 h; and (**g**–**i**) highly magnified SEM images of cells after culturing for 120 h.

In general, a polygonal shape for cells represents an improvement of cell conditions^30^. Once in contact with an implant, cells explore it using filopodia, lamellipodia and peripheral ruffles, which contribute to polarised edges. After cell seeding and culturing for 120 h, SaSO2 cells displayed full spreading with a spindle-shaped morphology on the surfaces of Mg1Zr2Sr3Ho (Fig. 5e). Compared to the other Mg-Zr-Sr-Ho alloys, Mg1Zr2Sr3Ho exhibited desirable morphology, attachment and spreading of the cultured SaSO2, as indicated by flourishing growths of filopodia with widely extended microspikes solidly attached to the surface of the alloy (highly magnified SEM, Fig. 5h). In contrast to Mg1Zr2Sr3Ho with high proliferation of SaSO2 cells, Mg1Zr2Sr1Ho displayed the lowest cell viability after culturing for 120 h (Fig. 5d,g). In the case of Mg1Zr2Sr5Ho, although some SaOS2 cells on its surface displayed shrinking-shaped rounds without long filopodia after seeding and culturing for 120 h (Fig. 5f), Mg1Zr2Sr5Ho still exhibited superior cell proliferation to Mg1Zr2Sr1Ho in terms of cell spread and viability.

**Figure 6:**
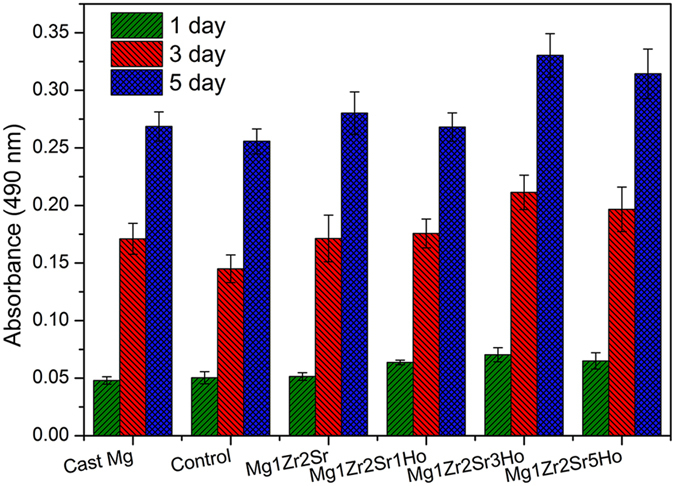
Cell numbers on Mg-Zr-Sr-Ho alloys after incubation for various periods.

The *in vitro* cytotoxicity of the Mg-Zr-Sr-Ho alloys was assessed using osteoblast-like cells (SaOS2). The optical densities in the extract of each alloy were measured using MTS assay and are listed in Fig. 6. The optical density measured by MTS assay is proportional to the number of cell. The control group was regarded as biocompatible. A higher optical density of an Mg alloy compared with that of control group represents a lower cytotoxicity. After culturing for 24 h, the cytotoxicity of Mg1Zr2Sr3Ho was slightly lower than that of the other Mg-Zr-Sr-Ho alloys, which exhibited a similar level of cytotoxicity to cast Mg and the control group. After culturing for 72 h, a remarkable difference in the cytotoxicity of the alloys was characterized. Specifically, the Mg-Zr-Sr-Ho alloys displayed relatively lower cytotoxicity compared to pure Mg, Mg1Zr2Sr and the control group, indicating that the addition of Ho (≤5%) to Mg alloys enhanced the growth of SaOS2 cells. After culturing for 120 h, Mg1Zr2Sr3Ho and Mg1Zr2Sr5Ho still maintained lower cytotoxicity than the other groups. The results of the *in vitro* cytotoxicity assessment of Mg-Zr-Sr-Ho alloys offer evidence that their cytotoxicity is associated with their corrosion rate: a lower corrosion rate always denotes a lower cytotoxicity.

## Discussion

In this study, an increase in Ho addition significantly enhanced the compressive yield strength (Fig. 4a) and the ultimate compressive strength, but reduced the ultimate strain (Fig. 4b) of the Mg-Zr-Sr-Ho alloys, which is in agreement with the conclusions of previous studies on the effects of REEs on the mechanical properties of Mg alloys^31^,^32^,^33^. The results of this study indicate that the ultimate compressive strength was significantly improved and the ultimate strain decreased with increasing Ho addition from 1% to 5% (Fig. 4). This is due to the following two reasons. First, the volume fraction of the Ho-containing intermetallic phases (MgHo_3_ and Mg_2_Ho) increased with an increase in the Ho of the Mg alloys. The intermetallic phases have inherent high hardness and strength, so high volumes of Mg-Ho phases will cause high strength. Furthermore, in this study the MgHo_3_ and Mg_2_Ho phases were mainly distributed along the grain boundaries. This may restrain the progress of the deformation during loading-deformation and can lead to an increase in ultimate compressive strength but decreased ultimate strain^34^. Second, in the Mg-Zr-Sr-Ho alloys, Ho distributed not only along the grain boundaries, but also in the interiors of the Mg matrix (EDX mapping, Fig. 1). As a result, Ho addition might contribute to the solid solution strengthening because of the significant difference in atomic radius between Mg (1.73 Å) and Ho (2.3 Å), as a large difference in atomic radius would lead to substantial lattice distortion and enhance the solid solution strengthening of Mg alloys^35^.

In this study, the Mg-Ho phases were identified as MgHo_3_ and Mg_2_Ho, which differs from the reported Mg-Zn-Ho phases^15^ and the Mg_24_Ho_5_ phase along the grain boundaries^36^. An isolation between Sr and the Mg matrix was introduced with the increased addition of Ho (EDX mapping, Fig. 1), which weakened the galvanic effects, and reduced the corrosion rates of the Mg-Zr-Sr-Ho alloys. The effect of Ho on the corrosion behaviour of Mg-Zr-Sr-Ho alloys in most cases depends on the composition of the alloys, and the volume fraction and the distribution of the intermetallic phases. This can also be seen in the changes in the corrosion potential of the alloys after immersion in SBF for 2 h (Fig. 2a). Due to the addition of Ho, the corrosion potential of Mg1Zr2Sr, 1.6345 V_SCE_, decreased to −1.6401 V_SCE_ for Mg1Zr2Sr1Ho. This suggests that the Mg_17_Sr_2_ and Mg-Ho phases have different thermodynamic stability. The addition of Ho to Mg alloys may eliminate the galvanic effects between the Mg matrix and the other intermetallic phases, and thus can enhance the corrosion resistance.

In addition to the effect of the Mg-Ho phases, the impurities of Mg-Zr-Sr-Ho alloys may also influence the corrosion rates. Zhou *et al*.^37^ found that the addition of Ho to AZ91D alloys resulted in the formation of an AlMnHo phase; this newly formed phase not only reduced the volume fractions of Mg-Al phases, but also eliminated the effects of impurities on the corrosion of Mg alloys. As can be seen from Table S1, the Mg alloys contained Fe, Al, Mn and Si. Although Mn has been considered to be beneficial in relation to the transformation of Fe and other impurities into harmless intermetallic phases, the relatively higher concentrations of Fe (0.05 wt. %) in Mg1Zr2Sr1Ho can still deteriorate the corrosion resistance, as traces of Fe (≥0.003 wt. %) are detrimental to the corrosion resistance of Mg alloys^38^.

The utilisation of Ho in clinical applications mainly relates to the controllable radioactivity of the isotope element^39^,^40^ and the wide application of Ho in the treatment of tumors^41^,^42^ and has also made contributions to the treatment of cancer in human using Ho-doped microspheres with acceptable biocompatibility for the desired curative effects^43^,^44^ by controlling the concentration of Ho *in vivo*^45^. Given the popularity of using REEs in Mg alloys, researchers have explored Mg-Ho alloys in order to obtain desirable qualities of acceptable corrosion resistance, suitable mechanical properties and excellent curative effects^36^,^37^,^46^,^47^,^48^,^49^ Nevertheless, the biocompatibility of Ho, and in particular its biological role in Mg-Ho based alloys as hard tissue implants, has not been covered in these studies.

In this study, the biocompatibility of Ho has been explored by comparing the cytotoxicity and the performance of SaOS2 cells seeded on the Mg-Zr-Sr-Ho alloys. Compared with the control group, Ho addition to Mg1Zr2Sr boosted cell growth and proliferation. In particular, the cytotoxicity and cell adhesions of Mg-Zr-Sr-Ho alloys were dependent on Ho concentration. More specifically, the cell on Mg1Zr2Sr3Ho which exhibited the slowest corrosion rate among the Mg-Zr-Sr-Ho alloys were much higher in number than those of the other alloys after culturing for 120 h. This suggests that the dose of Ho is critical for designing and preparing Ho-containing Mg alloys, as a high dose of Ho has been demonstrated to have negative effects on biocompatibility^50^. In another study^51^ rats fed with Ho chloride in their diet at doses up to 500 mg (kg day)^−1^ for more than 2 months showed no adverse effect. Although the present study does not provide accurate Ho concentrations in relation to toxicity, it suggests that Ho is a biocompatible element and can be used as one of the elements in Mg alloys for hard tissue engineering by carefully selecting its concentration (≤5 wt. %) and monitoring the corrosion rates of Mg-Ho based alloys, because the dissolution of Mg alloys is always accompanied by the release of ions into the body fluid. Rapid corrosion rate of an Mg alloy may release an overdose of metallic ions which simultaneously affects tissue growth and cell proliferation^4^.

Rapid corrosion of Mg alloys always gives rise to more corrosion products. Thus, Mg alloys with different corrosion rates exhibit various surface conditions in terms of topography and roughness^52^,^53^. A rough surface with defects, e.g., cracks on the surface of Mg alloys, does not provide an ideal substrate for cell growth and adhesion. As can be seen from Fig. 5a,d, Mg1Zr2Sr1Ho initially had a rough surface with many cracks after cell culturing for 24 h, and these cracks were dramatically enlarged after cell culturing for 120 h in extracts. Larger and deeper cracks allow more attack from the components of the extracts, leading to accelerated corrosion of the Mg matrix. Thus, the generated hydrogen gas and the volatile pH value adjacent to the surface of Mg alloys, along with the peeled-off substrate, further deteriorated the cell growth and adhesion (Fig. 5d,g).

**Figure 7:**
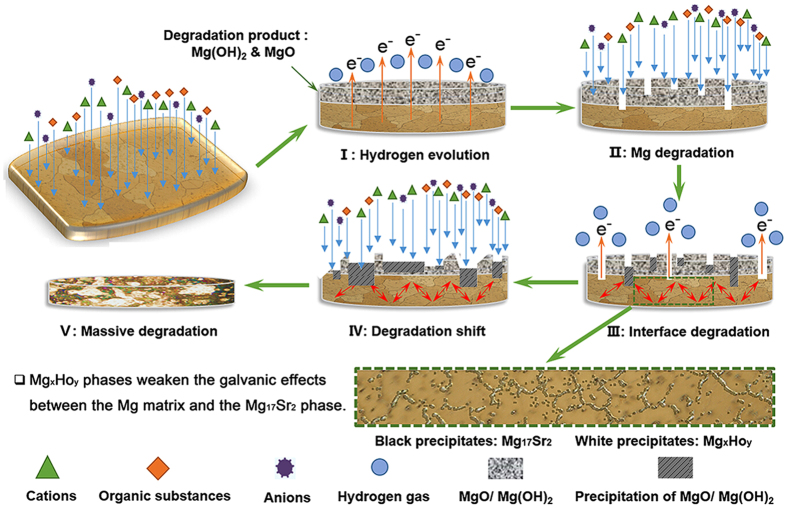


The corrosion process of Mg-Zr-Sr-Ho alloys in a physiological environment can be illustrated in five stages, as shown in Fig. 7 based on the results obtained in the present study.

### Stage I

Hydrogen evolution. Hydrogen evolution initiates at the interface between the Mg alloy and SBF due to the attack of components such as cations, organic substances and anions of the SBF, and Mg ions are released into the SBF so that an MgO/Mg(OH)_2_ layer is formed on the surface of the Mg alloys.

### Stage II

Mg degradation. As prolonged immersion time in SBF, some regions of the MgO/Mg(OH)_2_ layer convert to Mg^2+^ due to further attack from SBF (Fig. S2a,c). Consequently, the Mg substrate in these regions is exposed to the medium directly, leading to further degradation.

### Stage III

Interface degradation. As the degradation proceeds, more regions of the MgO/Mg(OH)_2_ layer are corroded constantly and more Mg substrates are exposed (Fig. S2c). Mg^2+^ may pass through the loosened MgO/Mg(OH)_2_ layer and form a new MgO/Mg(OH)_2_ layer on the exterior surface (Fig. 3g,h). Various components can also penetrate the loosened surface and attack the interior Mg substrate, leading to interface degradation between the Mg matrix and the components of the SBF beneath the surface layer. Meanwhile, the galvanic effects between Mg_17_Sr_2_ and the Mg matrix will be weakened due to the presence of the MgHo_3_ and Mg_2_Ho phases, which have a corrosion potential closer to that of the Mg matrix and are distributed along the grain boundaries.

### Stage IV

Degradation shift. The newly formed MgO/Mg(OH)_2_ layer cannot resist dissolution. Hence, uniform dissolution occurs and some regions are inevitably exposed to theSBF. The outer MgO/Mg(OH)_2_ layer is dissolved continuously and the corrosion extends to the interior Mg substrate and more regions would be exposed to the SBF.

### Stage V

Massive degradation. Some corroded residues may fall off the surface of Mg alloys, causing local pits in the Mg substrate and deeper cavities. Accordingly, the Mg matrix will be further attacked due to the galvanic effects and finally the Mg alloys will degrade completely in the SBF, resulting in the massive degradation.

This study has explicated a promising Mg alloy system (Mg1Zr2Sr3Ho) with excellent corrosion resistance compared to recently developed Mg alloys, such as Mg1Zr2Sr^19^, Mg-O alloys^54^ and coated Mg-Al based alloys^55^. Although the Mg1Zr2Sr3Ho alloy showed excellent performance in the assessment of cytotoxicity, there are still challenges in fully understand its biocompatibility. Particularly from the aspect of the development of biodegradable Mg alloy implants, it is essential to carry out thorough experimentation *in vivo* to provide accurate data on the safe dosage^3^ and biological properties, through taking advantage of new techniques such as the newly developed 3D printing of Mg alloys^56^, adjustment the phase constitutions^12^ and creative methods such as “materials smartness”^57^ and “bridge the gap between *in vitro* and *in vivo*”^58^.

## Methods

Mg1Zr2SrxHo alloys (x = 1, 3, 5 wt. %) were fabricated by melting of pure Mg, Mg-30Zr, Mg30Sr and Mg10Ho alloys (Hunan Rare Earth Metal Material Research Institute, China) with the designated compositions. The chemical compositions of the Mg alloys (Table S1) were determined by wavelength dispersion X-ray fluorescence (WDXRF, S4 Pioneer, Bruker, Germany). Samples with a diameter of 10 mm and thickness of 3 mm were further machined using electrical discharge machining (EDM) along the long axis of the Mg alloy ingots for microstructure characterisation, corrosion measurement and biocompatibility assessment. Cylindrical samples with a diameter of 5 mm and length of 10 mm were processed in the same way from the Mg alloy ingots for the compressive test.

Basic characteristics, including microstructure analysis, were investigated using optical microscopy (Olympus, DP70), scanning electron microscopy (SEM, Supra 55, Zeiss) equipped with energy-dispersive X-ray analysis (EDX) mapping technique, and X-ray diffraction (XRD-X’pert, Philips, The Netherlands) employing Cu-Kα radiation at an accelerating voltage of 40 kv and a current of 40 mA. To evaluate the effects of Ho addition on the mechanical properties of Mg alloys, compressive test was conducted on the Mg1Zr2SrxHo samples at an initial strain rate of 10^−3 ^s^−1^ using an Instron universal tester equipped with a video extensometer (Instron 5567, USA). The degradation behaviour of the Mg1Zr2SrxHo alloys was investigated using polarization measurement with a scan scope ±250 mv against open-circuit potential using a multichannel potentiostat (VSP 300, Bio-logic France), and immersion tests in SBF (the compositions are listed in Table S2), including hydrogen evolution, weight loss and characterization of degradation morphologies after immersion in SBF for various periods. For hydrogen evolution and weight loss, the SBF was refreshed every 4 h in order to maintain a stable pH value around 7.4. The hydrogen evolution rate *V*_*h*_ (ml cm^−2^ day^−1^) can be converted into the degradation rate *P*_*h*_ (mm year^−1^) using the equation given by *P*_*h*_ = 2.279V_h_^59^, and the corrosion rate (*Pw*, mm per year) based on weight loss was calculated from the weight loss rate *ΔW* (mg cm^−2^ day^−1^) using the equation *Pw* = 2.10 *ΔW*^60^.

To assess biocompatibility, SaOS2 cells were used to evaluate the cytotoxicity of the Mg alloys. These cytotoxicity assessments were carried out using extracts which were prepared using MEM α to extract Mg alloy specimens in a humidified atmosphere of 5% CO_2_ at 37 °C for 72 h (the details of the biocompatibility assessment are provided in the supporting information).

## Additional Information

**How to cite this article**: Ding, Y. *et al*. Mechanical properties, in vitro corrosion and biocompatibility of newly developed biodegradable Mg-Zr-Sr-Ho alloys for biomedical applications. *Sci. Rep.* **6**, 31990; doi: 10.1038/srep31990 (2016).

## Supplementary Material

Supplementary Information

## Figures and Tables

**Table 1:** Corrosion of Mg1Zr2Sr and Mg-Zr-Sr-Ho alloys in SBF.

Materials	*V*_*h*_ (ml cm^−2^ day^−1^)	*P*_h_^*^ (mm year^−1^)	*ΔW* (mg cm^−2^ day^−1^)	*P*_w_** (mm year^−1^)	*i*_*corr*_ (mA cm^−2^)	*P*_i_*** (mm year^−1^)
Mg1Zr2Sr1Ho	6.13 ± 0.14	13.96 ± 0.38	6.67 ± 0.14	14.02 ± 0.29	0.3290 ± 0.0011	7.52 ± 0.03
Mg1Zr2Sr3Ho	4.01 ± 0.09	9.15 ± 0.26	5.19 ± 0.16	10.90 ± 0.35	0.2712 ± 0.0017	6.19 ± 0.04
Mg1Zr2Sr5Ho	4.86 ± 0.14	11.08 ± 0.38	6.55 ± 0.15	13.76 ± 0.33	0.2928 ± 0.0010	6.69 ± 0.02
Mg1Zr2Sr	6.31 ± 0.11	14.39 ± 0.31	7.05 ± 0.17	14.80 ± 0.36	0.4996 ± 0.0182	11.42 ± 0.34

*Calculated based on H_2_ evolution; **calculated based on weight loss;***calculated based on potentiodynamic polarisation.
