# Supplementary material for: Mechanical properties, in vitro corrosion and biocompatibility of newly developed biodegradable Mg-Zr-Sr-Ho alloys for biomedical applications
Source: Sci Rep. 2016 Aug 24;6:31990. doi: 10.1038/srep31990 (PMC4995491; doi:10.1038/srep31990)
Supplement: Supplementary Information [file srep31990-s1.doc]

Mechanical properties, in vitro corrosion and biocompatibility of newly developed biodegradable Mg-Zr-Sr-Ho alloys for biomedical applications

Yunfei Ding^1^, Jixing Lin^2^, Cuie Wen^1^, Dongmei Zhang^3^, Yuncang Li^1,*^

^1^School of Engineering, RMIT University, Melbourne, Victoria 3001, Australia

^2^Department of Materials Science and Engineering, Jilin University, Changchun, Jilin 130025, China

^3^Institute for Frontier Materials, Deakin University, Geelong, Victoria 3217, Australia

**Supplementary Information**

**A. Biocompatibility assessment.** Human osteoblast-like cells (SaOS2) (Barwon Biomedical Research, Geelong Hospital, Australia) were used to evaluate the cytotoxicity of the Mg alloys and cultured in minimum essential medium alpha (MEM α, Gibco) supplemented with 10 % fetal bovine serum (FBS, Gibco) and 1 % penicillin/streptomycin in an incubator with humidified atmosphere of 5 % CO_2_ at 37 ^o^C. The cytotoxicity assessments were carried out using the indirect contact method^19^. Extracts were prepared using MEM α to extract Mg alloy specimens with a ratio (surface area of Mg alloys to volume of MEM α) of 0.8 cm^2^ ml^-1^ in a humidified atmosphere of 5 % CO_2_ at 37 ^o^C for 72 h. The extracts, after filtering using 0.22 μm filters, were used directly in the following MTS assay. A negative control was prepared through incubating MEM α in a humidified atmosphere of 5 % CO_2_ at 37 ^o^C for 72 hour. The SaOS2 cells were seeded in a 48-well plate with 400 μl MEM α supplemented with 10 % FBS and 1 % pencillin/streptomycin at a density of 1x10^4^ cells per well and incubated for 24 h to allow attachment on the plate. Then, the MEM α was replaced with 400 μl respective Mg alloy specimen extracts and control extracts. After culturing for 24, 72 and 120 h, the cell number in each well was measured using the MTS assay. In brief, the extract/control medium in each well was drawn out and washed three times with PBS. Then, 300 μl phenol red free RPMI 1640 medium (Gibco) was added to each well. After that, 100 μl MTS/PMS solution was added to each well, and the plate was incubated preventing from light for 1 h at 37 ^o^C. After incubation, 100 μl solution from each well was transferred into a 96-well plate. The optical density was measured by a microplate reader at 490 nm. The cell number is linear to the optical density.

**B. Cell adhesion Observation**. The Mg-Zr-Sr-Ho alloy samples for cell adhesion observation were placed in a 48-well plate. Each well containing a sample disc was seeded with 3 × 10^4^ of SaOS2 cells and incubated at 37 ^o^C in humidified air with 5% CO_2_ for 24 and 120 h, respectively. After incubation, samples were washed three times using PBS, and fixed in 3.9% glutaraldehyde solution for 20 min at room temperature and rinsed three times with PBS, followed by dehydration in an ethanol/distilled water gradient (50%, 60%, 70%, 80%, 90%, and 100%) for 10 min each. Then, the cells were chemically dried with 100 % hexamethyldisilazane (HMDS) for ten min. The HMDS was drained out and left the samples in a fumehood overnight to dry completely. Adherent SaOS2 cells on the surface of Mg1Zr2SrxHo alloy samples were gold-coated and observed by SEM (Supra 55, Zeiss, Germany).

**Table S1**. Chemical compositions of Mg-Zr-Sr-Ho alloys.

| Alloys | Chemical composition (wt. %) | | | | | | | |
| --- | --- | --- | --- | --- | --- | --- | --- | --- |
|  | Zr | Sr | Ho | Si | Al | Fe | Mn | Mg |
| Mg1Zr2Sr1Ho | 0.99 | 1.86 | 0.97 | 0.03 | 0.02 | 0.05 | 0.03 | Balance |
| Mg1Zr2Sr3Ho | 1.04 | 2.11 | 2.98 | 0.01 | 0.01 | 0.02 | 0.01 | Balance |
| Mg1Zr2Sr5Ho | 0.93 | 1.97 | 4.99 | 0.02 | 0.01 | 0.02 | 0.01 | Balance |
| Mg1Zr2Sr | 0.89 | 2.04 | N/A | 0.02 | 0.03 | 0.01 | 0.01 | Balance |

**Table S2**. Recipe for preparing m-SBF for a total volume of 1000 ml SBF.

| Reagent | Order | Amount |
| --- | --- | --- |
| NaCl | 1 | 5.403 g |
| NaHCO_3_ | 2 | 0.504 g |
| Na_2_CO_3_ | 3 | 0.426 g |
| KCl | 4 | 0.225 g |
| K_2_HPO_4_ 3H_2_O | 5 | 0.230 g |
| MgCl_2_ 6H_2_O | 6 | 0.311 g |
| 0.2 M NaOH | 7 | 100 ml |
| HEPES | 8 | 17.892 g |
| CaCl_2_ | 9 | 0.293 g |
| Na_2_SO_4_ | 10 | 0.072 g |
| 1.0 M NaOH | 11 | ≈ 15 ml |


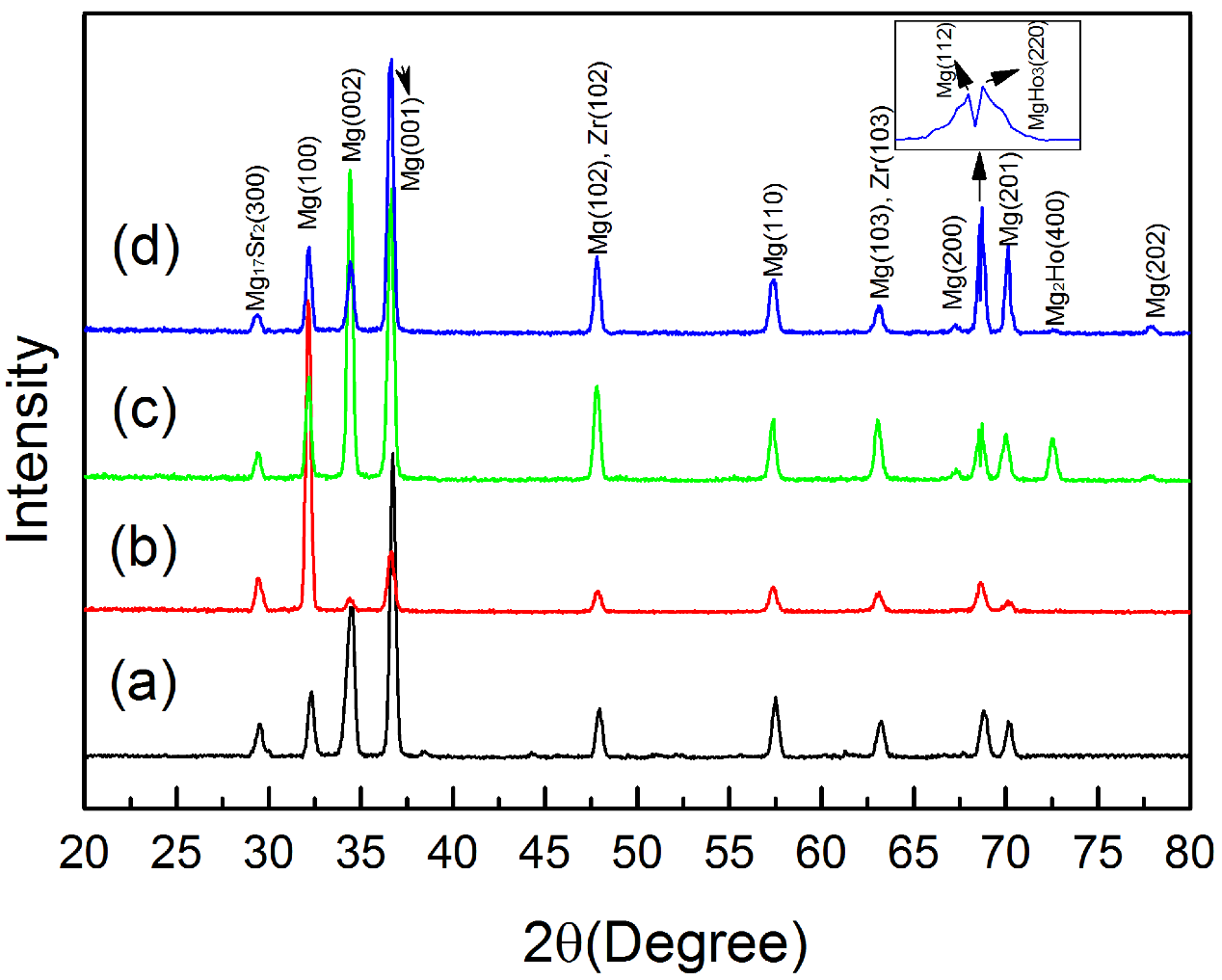


**Figure S1**. XRD patterns of Mg-Zr-Sr-Ho alloys before immersion tests: (a) Mg1Zr2Sr, (b) Mg1Zr2Sr1Ho, (c) Mg1Zr2Sr3Ho, (d) Mg1Zr2Sr5Ho.


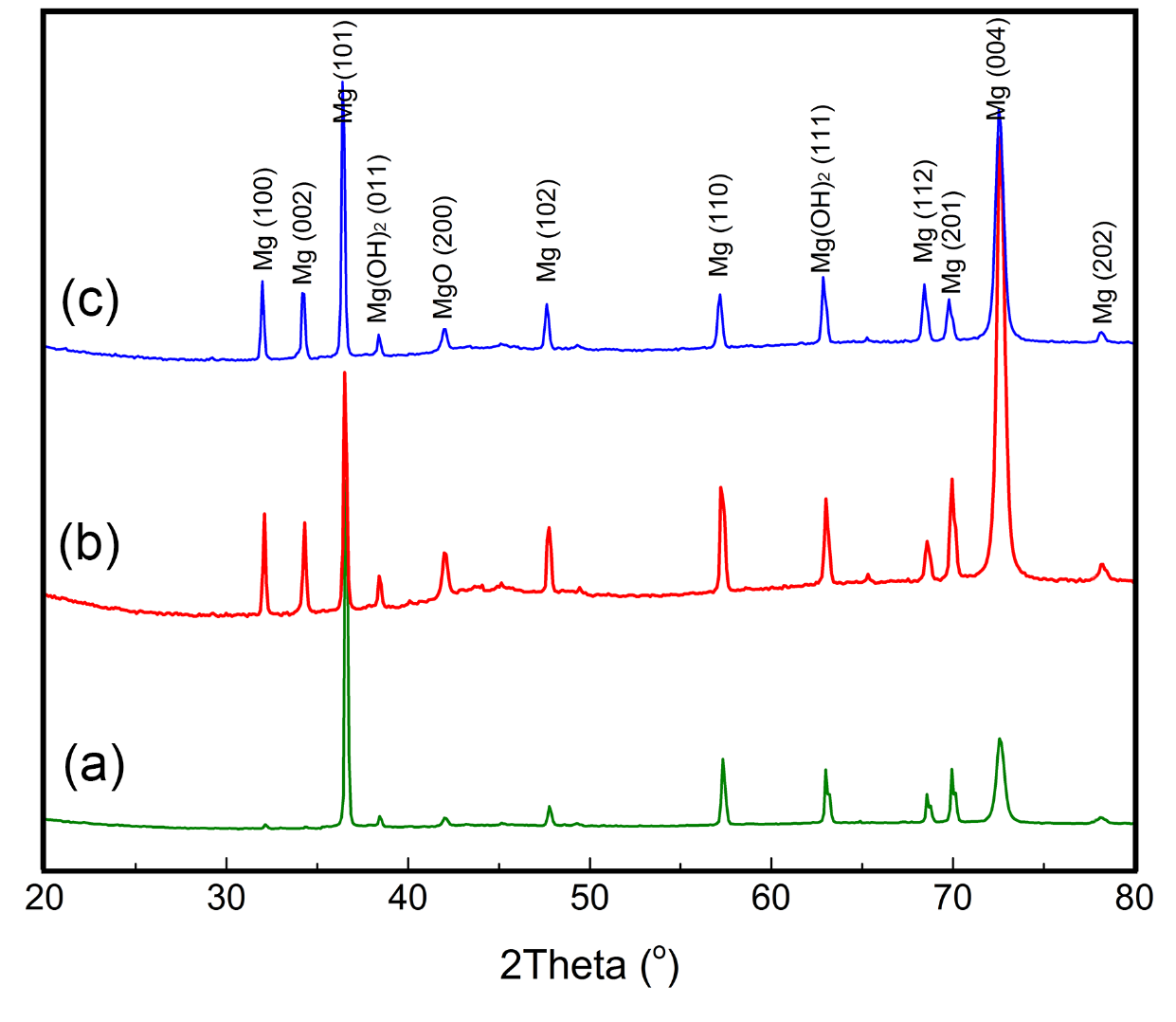


**Figure S2**. XRD patterns of Mg-Zr-Sr-Ho alloys after 24 h immersion in SBF: (a) Mg1Zr2Sr1Ho, (b) Mg1Zr2Sr3Ho, (c) Mg1Zr2Sr5Ho.
